# Proteomic Analysis of Legume–Microbe Interactions

**DOI:** 10.1002/cfg.263

**Published:** 2003-04

**Authors:** Barry G. Rolfe, Ulrike Mathesius, Michael Djordjevic, Jeremy Weinman, Charles Hocart, Georg Weiller, W. Dietz Bauer

**Affiliations:** 1 Genomic Interactions Group The Australian National University GPO Box 475 ACT Canberra 2601 Australia; 2 Ohio State University OH USA


**Comparative and Functional Genomics**
*Comp Funct Genom* 2003; **4**: 225–228.
Published online 1 April 2003 in Wiley InterScience (www.interscience.wiley.com). **DOI:** 10.1002/cfg.263

**Conference Review**

### Proteomic analysis of legume–microbe interactions

Barry G. Rolfe[1]*, Ulrike Mathesius[1], Michael Djordjevic[1], Jeremy Weinman[1], Charles Hocart[1], Georg Weiller[1] and W. Dietz Bauer[2]

[1] *Genomic Interactions Group, The Australian National University, Canberra, ACT, Australia*
[2] *Ohio State University, OH, USA*

**Correspondence to:*
*Barry G. Rolfe, Genomic Interactions Group, The Australian National University, GPO Box 475, Canberra, ACT 2601, Australia.*
*E-mail: rolfe@rsbs.anu.edu.au*

Received: 28 January 2003
Accepted: 4 February 2003

**Keywords: proteomics; peptide mass fingerprinting; legume–*Rhizobium* symbiosis; *Sinorhizobium meliloti*; *Medicago truncatula***

### Introduction

Legumes are very special plants. They form symbioses not formed by most other plants, including *Arabidopsis*, and are important sources of protein for animals and humans. Legume roots are invaded and colonized by the nitrogen-fixing soil bacteria called rhizobia [29], and also with mycorrhizal fungi which contribute to plant phosphorus acquisition [16]. The development of the root nodule meristem is unique, as its site, timing of initiation, the target cell type and the ontogeny can be defined. The elicitor (the nod factor) for cell division is known and can be synthesized. The beauty of the interaction is that the precise cell biology changes have been documented and many bacterial and some plant genes affecting nodulation have been characterized. In addition, mutants are available in both symbiotic partners. Within the nodule the nitrogen-fixing rhizobia, known as bacteroids, are surrounded by a host-derived membrane called the peribacteroid membrane (PBM) which controls molecular exchanges between the bacteroid and the legume cell [26]. The elicitor, or *Rhizobium* nod factor responsible for nodule initiation, is a lipochitin oligosaccharide [LCO] and plays a pivotal role in the induction of symbiotic developmental responses in legumes, leading to the formation of a nodule [29].

Proteomics is an ideal tool for the dissection of plant microbe interactions. First, it provides a broad overview of the proteins produced by both partners during their constant signal exchange and, in particular, it enables the effect on gene product networks of gene knockouts, additions and specific growth states to be determined. Second, it allows the detection of signal transduction pathways by following phosphorylation changes of proteins [27] that are important for protein function. The recent discovery of several plant receptor kinases responsible for the early detection and signal transduction of Nod factor perception [10,30] and autoregulation of nodule numbers [17,23,28], suggests that many early plant–microbe signalling events are regulated by phosphorylation events and key receptor kinases.

### Current model systems

To advance the analysis of plant microbe interactions, several model organisms have been chosen

Copyright © 2003 John Wiley & Sons, Ltd.

which provide either genomic or EST sequence information, a prerequisite for large-scale protein identification by peptide mass fingerprinting [7]. The two plants chosen are *Medicago truncatula* and *Lotus japonicus*, which have EST databases with about 180 000 and 32 000 entries (as of January 2003), respectively, and genome sequencing is under way for both species. Proteomic analysis has mainly focused on *M. truncatula*, for which a proteome reference map has been established [18; **http://semele.anu.edu.au/2d/2d.html**]. The model bacterial symbiont is *Sinorhizobium meliloti*, the symbiont of both *M. truncatula* and its relative alfalfa.

### Adaptations for survival and rapid recovery

*Rhizobium* bacteria exhibit several different lifestyles, in the soil environment, in the root–soil interface [rhizosphere] and within the root nodule. Microorganisms have evolved many mechanisms that enable them to rapidly meet changes in their environment. Resilience to these changes is essential to their survival, and depends on rapid and efficient control of genetic expression and metabolic responses [24]. The pathways that govern these responses are complex, often overlapping and, in general, poorly understood. There is little knowledge of how *S. meliloti* inhabits and flourishes in the rhizosphere and inside 'infection threads'. Nutrient availability in the rhizosphere and infection threads undergoes considerable qualitative, spatial and temporal variation, and rhizobia have evolved mechanisms involving multiple changes in gene expression to adapt to these changes [1]. Until recently, it has been difficult to isolate the rhizobia from these niches to study their particular cellular adaptations. However, microhabitat reproduction in the laboratory and microdissection, together with proteomics, now provide approaches to analyse these growth stages.

### Proteome studies of legume–microbe interactions

Three recent studies using proteomic analysis have examined different aspects of the *Rhizobium*–legume interaction. Natera *et al.* [22] compared the free-living bacterium grown in laboratory culture with the bacteroid form isolated from root nodules. Bestel-Corre *et al.* [2] used a time-course analysis of root protein profiles to study *Medicago truncatula* inoculated with either *S. meliloti* or with the arbuscular mycorrhizal fungus *Glomus mosseae*. Morris and Djordjevic [21] examined the proteome changes in a cultivar-specific legume–*Rhizobium* interaction. These studies have contributed to the description of the substantial changes that occur both within the bacterium grown under different circumstances and the temporal changes within the inoculated plant. A fourth research program investigated the proteins of the peribacteroid membrane (PBM) of soybean nodule bacteroids and their possible involvement in protein processing and the biogenesis and function of the PBM [26]. The proteomes and the distribution of the proteins of several legumes, including *M. truncatula, Melilotus alba* and *Trifolium subterraneum* were compared [19]. Protein identification, however, was most efficient in *M. truncatula* due to the existence of a large EST database necessary for peptide mass fingerprinting [18].

### A detailed proteome analysis of *Sinorhizobium meliloti* strain 1021

The *S. meliloti* genome consists of a 3.7 Mb chromosome and two megaplasmids of 1.4 and 1.7 Mb. The genome sequence is predicted to contain 6294 protein coding frames and has provided a better understanding of the possible functions of *S. meliloti* [11]. However, the gene sequence alone often reveals little about the function of the gene products. Thus, functional proteomics is beginning to play a role in the identification and analysis of gene networks at the level of protein expression [25]. Early studies established 2-DE as a reproducible tool for the display of over 2500 *S. meliloti* proteins [4,5,14] and used proteome analysis to discover flavonoid-induced proteins [12], plasmid-encoded functions important in symbiosis [13]. Proteome analysis was also used to demonstrate that a single mutation results in multiple protein changes in *S. meliloti* [15]. A more detailed proteomic examination of *S. meliloti* strain 1021 grown under a variety of growth conditions was recently described by Djordjevic and co-workers

Copyright © 2003 John Wiley & Sons, Ltd.

[8,9], using a combination of 2D gel electrophoresis, peptide mass fingerprinting and bioinformatics. This work was aided by the earlier development of a specialized proteomic database for comparing matrix-assisted laser desorption/ionization–time of flight mass spectrometry data of tryptic peptides with corresponding sequence database segments [32]. Djordjevic and co-workers [8,9,32] identified the protein products of 810 genes (13.1% of the genome's coding capacity) and the activity of 53 metabolic pathways. Other proteins representing ABC-type transporters, membrane and regulatory proteins, nodule-specific proteins, and nutrient stress-specific proteins were identified. This information was used to describe the processes occurring in *S. meliloti* cells in nodules and under various stress conditions. This work demonstrated the utility of combining mass spectrometry with protein arraying to identify candidate genes involved in important biological processes and the occupation of niches that may be intransigent to other methods of gene expression profiling.

### *Rhizobium* makes a series of extracellular N-acyl homoserine lactone signals

In Gram-negative bacteria many important changes in gene expression and behaviour, such as the synthesis of exoenzymes, exopolysaccharides and the colonizing of hosts, are regulated in a population density-dependent manner by N-acyl homoserine lactone [AHL] molecules called 'quorum sensing signals' [31]. The synthesis of AHL signals is common among plant-associated bacteria and probably plays a central role in ecological interactions amongst microbial communities and between bacteria and their eukaryotic hosts [3]. Proteome analysis was used to show that the eukaryotic host, *M. truncatula*, was able to detect nanomolar to micromolar concentrations of bacterial AHLs from both symbiotic (*S. meliloti*) and pathogenic (*Pseudomonas aeruginosa*) bacteria [20]. *M. truncatula* responded in a global manner with significant changes in the accumulation of over 150 proteins. The accumulation of specific proteins and isoforms depended on AHL structure, concentration and time of exposure. In addition, exposure to AHLs was found to induce changes in the secretion of compounds by the plants that mimic quorum-sensing signals and thus have the potential to disrupt quorum sensing in associated bacteria.

### What has proteomics contributed to the study of legume–microbe interactions?

So far, we are at the beginning of the proteome analysis of plant microbe interactions. Major advances have been made in the microbial partner, partly because of the ease of culturing and the fact that it is a single cell, rather than a multicellular organism, but also because a complete genome sequence is available, making the use of peptide mass fingerprinting highly successful [9]. The focus so far has been on the new discovery of proteins involved in symbiosis, some of their post-translational modifications, identification of specific isoforms of proteins involved in certain pathways, and the construction of biochemical pathways in which the discovered proteins act. The trend has gone from the initial protein identification by N-terminal sequencing [21,22,26] to large-scale protein identification using peptide mass fingerprinting [9,18]. Advances will need to be made in subcellular fractionation, protein resolution and recovery of low-abundance, hydrophobic and integral membrane proteins. The use of LC–MS/MS and the development of more sensitive mass spectrometers is likely to solve some of the current problems by allowing separation of proteins undetectable on 2-DE gels as well as the analysis of protein complexes. Combining these techniques with rigorous biochemical characterization of protein function, as well as comparing the data to other currently used post-genomic techniques [6] will make the use of proteomics more functional in the legume–microbe interaction field.

### Acknowledgements

Part of this work was supported by a block grant from the Australian Government to the Australian National University and the ARC Discovery Grant DP 0209075 to U.M.

### References

1. Andrews JH, Harris RF. 2000. The ecology and biogeography of microorganisms of plant surfaces. *Ann Rev Phytopathol* **38**: 145–180.

Copyright © 2003 John Wiley & Sons, Ltd. 

2. Bestel-Corre G, Dumas-Gaudot E, Poinsot V, *et al.* 2002. Proteome analysis and identification of symbiosis-related proteins from *Medicago truncatula* Gaertn. by two-dimensional electrophoresis and mass spectrometry. *Electrophoresis* **23**: 122–137.
3. Cha C, Gao P, Chen YC, *et al.* 1998. Production of acyl-homoserine lactone quorum-sensing signals by gram negative plant-associated bacteria. *Mol Plant Microbe In* **11**: 1119–1129.
4. Chen HC, Higgins J, Kondorosi E, *et al.* 2000a. Identification of *nolR*-regulated proteins in *Sinorhizobium meliloti* using proteome analysis. *Electrophoresis* **21**: 3823–3832.
5. Chen HC, Higgins J, Oresnik IJ, *et al.* 2000b. Proteome analysis demonstrates complex replicon and luteolin interactions in *pSyma*-cured derivatives of *Sinorhizobium meliloti* strain 2011. *Electrophoresis* **21**: 3833–3842.
6. Colebatch G, Trevaskis B, Udvardi M. 2002. Symbiotic nitrogen fixation research in the postgenomics era. *New Phytologist* **153**: 37–42.
7. Cordwell SJ, Wilkins MR, Cerpapoljak A, *et al.* 1995. Cross-species identification of proteins separated by two-dimensional gel electrophoresis using matrix-assisted laser desorption ionization time-of-flight mass spectrometry and amino acid composition. *Electrophoresis* **16**: 438–443.
8. Djordjevic MA, Natera SH, Chen H-C, *et al.* 2002. Proteome analysis of *Sinorhizobium meliloti*. In *Nitrogen Fixation, Global Perspectives*, Finan T, O'Brian M, Layzell D, Vessey K, Newton W (eds). CABI: New York; 50–54.
9. Djordjevic MA, Weiller G, Chen H-C, *et al.* 2003. A proteomic approach for discovery of new genes for nodule occupancy and stress adaptation in the model symbiont *Sinorhizobium meliloti*. *Mol Plant Microbe In* (in press).
10. Endre G, Kereszt A, Kevei Z, *et al.* 2002. A receptor kinase gene regulating symbiotic nodule development. *Nature* **417**: 962–966.
11. Galibert F, Finan TM, Long SR, *et al.* 2001. The composite genome of the legume symbiont *Sinorhizobium meliloti*. *Science* **293**: 668–672.
12. Guerreiro N, Redmond JW, Rolfe BG, Djordjevic MA. 1997. New *Rhizobium leguminosarum* flavonoid-induced proteins revealed by proteome analysis of differentially displayed proteins. *Mol Plant Microbe In* **10**: 506–516.
13. Guerreiro N, Stepkowski T, Rolfe BG, Djordjevic MA. 1998. Determination of plasmid-encoded functions in *Rhizobium leguminosarum* biovar *trifolii* using proteome analysis of plasmid-cured derivatives. *Electrophoresis* **19**: 1972–1979.
14. Guerreiro N, Djordjevic MA, Rolfe BG. 1999. Proteome analysis of the model microsymbiont *Sinorhizobium meliloti*: isolation and characterization of novel proteins. *Electrophoresis* **20**: 818–825.
15. Guerreiro N, Ksenzenko VN, Djordjevic MA, *et al.* 2000. Elevated levels of synthesis of over 20 proteins results after mutation of the *Rhizobium leguminosarum* exopolysaccharide synthesis gene *pssA*. *J Bacteriol* **182**: 4521–4532.
16. Harrison MJ. 1999. Molecular and cellular aspects of the arbuscular mycorrhizal symbiosis. *Ann Rev Plant Physiol* **50**: 361–389.
17. Krusell L, Madsen LH, Sato S, *et al.* 2002. Shoot control of root development and nodulation is mediated by a receptor kinase. *Nature* **420**: 422–426.
18. Mathesius U, Keijzers G, Natera SHA, *et al.* 2001. Establishment of a root proteome reference map for the model legume *Medicago truncatula* using the expressed sequence tag database for peptide mass fingerprinting. *Proteomics* **1**: 1424–1440.
19. Mathesius U, Imin N, Chen HC, *et al.* 2002. Evaluation of proteome reference maps for cross-species identification of proteins by peptide mass fingerprinting. *Proteomics* **2**: 1288–1303.
20. Mathesius U, Mulders S, Gao M, *et al.* 2003. Extensive and specific responses of a eukaryote to bacterial quorum sensing signals. *Proc Natl Acad Sci USA* (in press).
21. Morris AC, Djordjevic MA. 2001. Proteome analysis of cultivar-specific interactions between *Rhizobium leguminosarum* biovar *trifolii* and subterranean clover cultivar Woogenellup. *Electrophoresis* **22**: 586–598.
22. Natera SHA, Guerreiro N, Djordjevic MA. 2000. Proteome analysis of differentially displayed proteins as a tool for the investigation of symbiosis. *Mol Plant Microbe In* **13**: 995–1009.
23. Nishimura R, Hayashi M, Wu G-J, *et al.* 2002. HAR1 mediates systemic regulation of symbiotic organ development. *Nature* **420**: 426–429.
24. Nystrom T. 1998. To be or not to be: the ultimate decision of the growth-arrested bacterial cell. *FEMS Microbiol Rev* **21**: 283–290.
25. Pandey A, Mann M. 2000. Proteomics to study genes and genomes. *Nature* **405**: 837–846.
26. Panter S, Thomson R, de Bruxelles G, *et al.* 2000. Identification with proteomics of novel proteins associated with the peribacteroid membrane of soybean root nodules. *Mol Plant Microbe In* **13**: 325–333.
27. Peck SC, Nuhse TS, Iglesias A, *et al.* 2001. Directed proteomics identifies a plant-specific protein rapidly phosphorylated in response to bacterial and fungal elicitors. *Plant Cell* **13**: 1467–1475.
28. Searl IR, Men AE, Laniya T, *et al.* 2002. Long-distance signalling in nodulation directed by a CLAVATA1-like receptor kinase. *Science* **299**: 109–112.
29. Spaink HP. 1996. Regulation of plant morphogenesis by lipochin oligosaccharides. *Crit Rev Plant Sci* **15**: 559–582.
30. Stracke S, Kistner C, Yoshida S, *et al.* 2002. A plant receptor-like kinase required for both bacterial and fungal symbiosis. *Nature* **417**: 959–962.
31. Teplitski M, Robinson JB, Bauer WD. 2000. Plant secrete substances that mimic bacterial bacterial N-acyl homoserine lactone signal activities and affect population density-dependent behaviors in associated bacteria. *Mol Plant Microbe In* **13**: 637–648.
32. Weiller GF, Djordjevic MA, Caraux G, *et al.* 2001. A specialised proteomic database for comparing matrix-assisted laser desorption/ionization–time of flight mass spectrometry data of tryptic peptides with corresponding sequence database segments. *Proteomics* **1**: 1489–1494.

Copyright © 2003 John Wiley & Sons, Ltd. 
